# The Vaccination Question

**Published:** 1895-03-23

**Authors:** 


### MEDICAL PROGRESS AND HOSPITAL CLINICS.

[*The Editor will be glad to receive offers of co-operation and contributions from members of the profession. All letters should be addressed to* THE EDITOR, THE LODGE, PORCHESTER SQUARE, LONDON, W.]

### THE VACCINATION QUESTION.

(*Continued from page* 422.)

STILL criticising the statistical methods of those who support vaccination, Mr. Hutton[1] refers to the late Professor Guy, of King's College, and as that writer is unfortunately no longer alive to defend himself, while his paper on the subject is buried in the Transactions of the Statistical Society, it may be as well to clear him from the misrepresentation which he suffers at the hands of Mr. Hutton, especially as the question with which Dr. Guy deals is of some interest. Mr. Hutton says (p. 57): "The late Dr. Guy, who read a paper on 'Two Hundred and Fifty Years of Small-pox in London' before the Statistical Society on June 20th, 1882, proved to his own satisfaction, and doubtless to that of his hearers, that, thanks to vaccination, there had been no epidemic of small-pox at all in London during the nineteenth century; and this was done by assuming an epidemic to mean 10 per cent. of the deaths from all causes being due to the one particular disease in question, whereas in the year 1871 the ratio was only 9·837 per cent. ('Journal of the Statistical Society,' Vol. XLV., p. 404). The paper is a very laborious one, and contains much valuable information; but when a writer plainly asserts (p. 414) that the question of the preventive power of vaccination is exclusively one of statistics, thus leaving out of account altogether, although himself a physician, the pathological side of the subject, which is more strictly allied to medical science, we have a right to expect that he shall not use the figures thus arbitrarily." Turning now to Guy's paper, we find that this is how he deals with the matter: "Though we understand what an epidemic malady means, and know that it implies a considerable and irregular excess of attacks and deaths in certain years as compared with others, and though, as an example in point, we should speak of small-pox as epidemic and pulmonary consumption as non-epidemic, we are not able to state with numerical precision what amount of fluctuation or difference from year to year shall be held to constitute an epidemic, or what multiple of the average or minimum of deaths. In the absence of any numerical rule applicable to this subject, we shall not go far wrong, if, passing in review the figures relating to a term of years, we lay hold of those that stand out in relief (if I may so express myself) from the figures which precede and follow. In the case of small-pox, I find such convenient figures in all numbers exceeding 100 (*i.e.*, 100 deaths from small-pox in 1,000 from all causes), and, with a view to a comparison of one period with another, I name 100 deaths or upwards an epidemic." Having thus stated his own view, and given some relative figures, Dr. Guy continues as follows: "Lest, however, this selection of a particular ratio should be deemed arbitrary, and possibly misleading, I construct a table in which I display side by side the number of years in which the ratios exceed the several figures 50, 75, 100, and 150 per 1,000."

TABLE II.

| Ratios. | Centuries. | | |
|---|---|---|---|
| | Seventeenth. | Eighteenth. | Nineteenth. |
| 50 per 1,000 and less than 75 | 11 (48 yrs.) | 22 (100 ys.) | 10 (72 yrs.) |
| 75 ,, 100 | 4 ,, | 27 ,, | 4 ,, |
| 100 ,, 150 | 10 ,, | 29 ,, | 0 ,, |
| 150 and upwards 0 | 0 ,, | 4 ,, | 0 ,, |
| 50 and upwards ... ... | 25 | 62 | 14 |

Having discussed this table in detail, Dr. Guy gives another, in which he shows the maxima and minima of prevalence in the seventeenth, eighteenth, and nineteenth centuries respectively:—

TABLE III.

| Centuries. | Maxima. | Minima. | Range. |
|---|---|---|---|
| Seventeenth century (48 years) | 124·40 | 2·98 | 121·42 |
| Eighteenth ,, (100 ,, ) | 183·94 | 15·32 | 168 62 |
| Nineteenth ,, (72 ,, ) | 98·37 | 0·56 | 97·81 |

Further on in the paper he returns to the subject thus: "I have already stated that in no year of the present century has the ratio of deaths by small-pox to deaths by all causes attained the high level of 100 and upwards so frequent in the last years of the eighteenth. If we count the year 1800 as the last of the eighteenth century, these high ratios ended with that year, in which the ratio of deaths by small-pox was 10,443 per 100,000. Twice only in the 72 years of the present century did the ratio approximate at all closely to that high figure. These were the years 1805 and 1871, when the figures were 9,592 and 9,837 respectively. For the past year, 1881, they were 2,924. In 58 years, then, we had two epidemics approaching in severity the mildest of the old epidemics of the seventeenth and eighteenth centuries. In 37 years of the seventeenth century we had nine such epidemics; in 55 years of the eighteenth we had 24." These quotations show that Dr. Guy does not by any means confine himself or his readers to the "10 per cent." assumption of an epidemic, for he gives also the facts on the basis of 5 per cent., 7½ per cent., and 15 per cent. Moreover, he supplies the very figures which Mr. Hutton adduces in the extract above given from the work under review.

Of course this misrepresentation of Guy is quite unintentional on Mr. Hutton's part. But Guy, being a believer in vaccination is, in Mr. Hutton's estimation, untrustworthy, and, without taking the trouble to make himself acquainted with what he professes to criticise, Mr. Hutton proceeds to find fault with Guy, just as on the other hand he accepts Creighton and Crookshank without quarrel or question as to any single thing they choose to say. In this connection it is very odd to notice how Mr. Hutton deals with Guy and Mr. Russel Wallace. Mr. Hutton is of opinion that vaccination is much more than a statistical question, and in the passage above quoted he adversely refers to Guy as holding "that the question of the preventive power of vaccination is

[1] "The Vaccination Question." A letter addressed by permission to the Right Hon. H. H. Asquith, Q.C., M.P., by A. W. Hutton, M.A., Librarian to the National Liberal Club. (Methuen and Co., 36, Essex Street, W.C. Price 1s. 6d.)

exclusively one of statistics." For Wallace, however, Mr. Hutton has nothing but praise. At one time, misquoting that writer's essay as "Vaccination proved Useless and Dangerous from Twenty-five Years of Registration Statistics," he speaks of it as "an able and convincing criticism of the statistical arguments," and at another time, differently misquoting the same pamphlet as ". . . from Fifty-five Years . . ." &c. (the actual title being "Forty-five Years of Registration Statistics proving Vaccination to be both Useless and Dangerous"), he declares that the writer has given an explanation "disastrous to the trustworthiness" of certain pro-vaccinal figures. But he nowhere mentions that Wallace, whom he praises, urges quite as strongly as Guy the view on which he animadverts, namely, that (to quote Wallace) "the utility or otherwise of vaccination is purely a question of statistics." That kind of criticism is reserved for Guy, but surely if ever a man was justified in confining himself to the statistical aspect of the question, it was in a paper read to the Statistical Society, where "the pathological side of the subject" would have been absurdly out of place. Similarly, speaking of Mr. Crookshank, the Professor of Bacteriology in King's College, he describes him as "a medical scientist of European reputation." But, alas, when a bacteriologist like Klein has the audacity to publish investigations tending to support the view that vaccination prevents small-pox, we are told that "bacteriology is still in its infancy," and that "Dr. Klein's paper, so far from rehabilitating vaccination, will rather tend to discredit bacteriology as a method of diagnosis." In short, medical men who approve of vaccination are negatively ignorant or actively dishonest, while its opponents are unbiassed searchers after truth: statistics which support vaccination are untrustworthy, while statistics on the other side, no matter though in some directions they omit 50 per cent. of essential facts, are of "significance unmistakable," which only hopelessly prejudiced persons "can be blind to": and a bacteriologist who opposes vaccination is certified as of European reputation, while another who supports it only brings his science into contempt by such contumacious conduct.

We agree with the writer that vaccination is a scientific question. In these days there is a tendency for questions of every sort to be dragged into the vortex of party politics, and it strikes us as rather ominous that a pamphlet on this subject, written by the librarian to the National Liberal Club, should be addressed with permission to the Home Secretary of a Liberal Government. It would be deplorable if the subject of the value of vaccination as a small-pox prophylactic should, in the slightest degree, take on a party complexion. The question of where the rights of a parent end with regard to the vaccination of his own child, and of where the rights of the public begin with regard to their protection from possible small-pox infection, is no doubt political. Mr. Hutton, however, does not by any means confine himself to such points, but details to Mr. Asquith his views on the history and pathology of vaccination and small-pox, and it is largely on these views that his recommendations as to legislation are based. Mr. Asquith is generally credited with being a level-headed statesman. Whatever may be his views as to a question of expediency like the repetition of vaccination penalties, we cannot imagine that he will rush to any decision regarding the merits or demerits of vaccination itself, as a result of perusing the one-sided statements and ill-digested conclusions contained in Mr. Hutton's pages, and without weighing on his own account the final report of the Vaccination Commission, and the evidence on which that report is founded.

In a book which has neither index nor table of contents it is not easy to follow all that a writer has to say on any particular point. Near the beginning, however, we noted with satisfaction that, while decrying the opinion of ordinary medical practitioners as of little moment, Mr. Hutton specially excluded from this criticism "those who have the care of patients in small-pox hospitals, and the evidence of these doctors," he says, "deserves the closest attention." Having come to the last page, and musing a little on what had gone before, it struck us that we had somehow passed over unnoticed that part of the work discussing the evidence, statistical and other, furnished by small-pox hospital physicians. But on once more working our way from page to page, all we can find is a casual and incomplete reference to the question of the percentage of vaccinated to unvaccinated admissions to hospital. Neither age, incidence, nor death rates are attempted to be dealt with, and though he talks of giving the "closest attention" to "the evidence of these doctors," he never seriously recurs to the subject, excepting possibly where he suggests, as his manner is, that such evidence is bound to be biassed and untrustworthy. On the contrary, he deliberately selects for his medical guides, authors who, so far as their writings show, may never have had charge of a small-pox ward, and may not even have had that occasional experience of the disease which belongs to the family physician, and of which Mr. Hutton thinks so little. It would almost appear that the investigation of this subject could, from Mr. Hutton's point of view, be quite as well carried on by the man in the moon, so far as any intimate acquaintance with actual small-pox is concerned. Let Mr. Hutton follow his own advice. Let him go to to men who have had to fight small-pox face to face, day by day and night by night, in the wards at Highgate, or Homerton, or Fulham. Let him ask them what they think of vaccination, either as a protection to themselves and their families, or as the supreme and outstandiag factor in the recovery or death of those who occupy the hospital beds. Or, still better, let him (defying the law like a loyal anti-vaccinator) follow Professor Crookshank's prescription and get himself protected by small-pox inoculation; then, if he survive, let him take a three months' residence in a small-pox hospital, and watch there, from beginning to end, the course of the disease in the vaccinated and unvaccinated. He will then see how vaccinated recover and unvaccinated die, or how the former are unmarked and the latter are scarred and disfigured; while as to the re-vaccinated nurses, their immunity rom harm in the midst of concentrated infection will remind him of the children in the fiery furnace seven times heated, "upon whose bodies the fire had no power . . . nor the smell of fire had passed upon them." Then at last Mr. Hutton will be in a position to judge of the honesty or dishonesty of hospital statistics, and the bias or no bias of hospital doctors, and to contrast the hard facts of daily observation and experience with the nebulous theorisings and speculations of cloudland.

